# Establishment and application of information-based training and assessment platform for clinical nursing operation technology

**DOI:** 10.1186/s12912-022-01053-3

**Published:** 2022-10-18

**Authors:** Xing Zheng, Aixia Ma, Jingai Huang, Chunlan Liu

**Affiliations:** 1 Department of Medical Oncology, Qilu Hospital, Cheeloo College of Medicine, Shandong University, Jinan, China; 2 Department of Nursing, Qilu Hospital, Cheeloo College of Medicine, Shandong University, Jinan, China; 3 Nursing Theory and Practice Innovation Research Center, Cheeloo College of Medicine, Shandong University, Jinan, China

**Keywords:** Nursing, Operation technology, Information system, Training, Assessment

## Abstract

**Background:**

The paper version of the training assessment was time-consuming and labor-consuming. It is an inevitable trend to change the appraisal method utilizing information technology. This study aimed to realize convenient and rapid management of the whole process of clinical nursing operation technology through information-based training and assessment platform.

**Methods:**

Combined with the operation mode of clinical nursing operation skills and set the basic functions of the information platform of clinical nursing operation training and assessment, the information-based training and evaluation platform for clinical nursing operation skills was established. The platform was officially operated in a tertiary level A general hospital in Shandong Province in 2018.

**Results:**

The information-based training and assessment platform is composed of Management Center (Computer Terminal) and a client terminal (APP terminal). The computer terminal contains 11 modules, and the APP terminal contains 8 modules. By December 2020, a total of 12,619 nurses had completed the training in nursing operation skills, and a total of 11,986 nurses had completed the examination. The examination results of nursing operation skills of the same nurses in 2018 were significantly higher than those in 2017(P < 0.05), and the error rate was significantly lower (P < 0.05). From 2016 to 2020, the scores of nasal feeding, CPR, and respiratory airbag of N1 level nurses significantly increased after using the information-based training and Assessment Platform (P < 0.05).

**Conclusion:**

Based on the information terminal training assessment can realize the management of the whole process of clinical nursing operation technology training and assessment, which is better than the traditional method, and is a very practical and convenient clinical training and assessment method.

**Supplementary information:**

The online version contains supplementary material available at 10.1186/s12912-022-01053-3.

## Background

With the rapid development of network technology, the combination of information technology and education has opened up more communication channels and operating modes for teaching work. The structure, content, and methods of education and teaching are undergoing tremendous changes, and the clinical nursing teaching career needs constant renewal. Besides, in 2021 Dubé et al. used bibliometric analysis to assess trends in educational technology and it shows that mobile and analytic technology have shown consistent trends over time [1]. Therefore, to further promote the development of clinical nursing teaching reform and the consistency of scientific and technological progress. At present, many hospitals in China have established comprehensive training centers for clinical skills, to set up advanced teaching model equipment, computer system, medical teaching tools, multimedia equipment, etc. [2], and even rely on medical physiology and computer technology to create virtual reality simulation teaching technology. The advantages of virtual worlds for conducting clinical medical education and assessment include greater time cost-effectiveness and significantly improved theoretical knowledge such as cognitive outcomes, compared with body model-based simulations and face-to-face lectures [3]. Moreover, telehealth encompasses a broad variety of technologies and methods to deliver virtual medical, health, and education services [4]. However, the operation and assessment of the paper-based version are still basically carried out. The paper-based version requires manual timing, and the results need to be calculated and manually entered into the computer, and error analysis is time-consuming and labor-consuming, It is an inevitable trend to change the appraisal method utilizing information technology [5]. In 2018, our hospital took the lead in relying on modern information technology to develop and apply the training and assessment of the technical operation of nursing staff based on information technology. The platform can realize the automatic management of the whole process of nursing staff information, various operating standards, operating assessment plan, operating assessment process, operating assessment results, statistical analysis, etc., thus it can not only improve the efficiency of training, but also improve the level of clinical operation, reduce the rate of error and improve the quality of clinical nursing. The effect of the evaluation platform based on information terminal training was validated.

## Methods

### Establishment and application of information-based training and assessment platform for clinical nursing operation technology

In this study, the Clinical Nursing Operation Technology Information Platform is based on the hospital Microsoft Windows Mobile 5.0 Information Platform System for software development, to ensure the stability and security of the Server side, adopting Visual Basic NET language. The database adopts Microsoft R SQL Server Compact Edition 2.0. The ADO.NET in Net CF acts as a data connection, using an android mobile handheld smart terminal. The basic functions of the platform include an information base for all nurses and assessment teachers, Operation Skill Assessment Standard Database, assessment process and result records, information statistical analysis, performance review, and administrator database, etc.

Clinical nursing operation technology information platform includes management center (computer terminal) and client terminal(APP end). (1) Computer side: According to different levels of administrators to implement hierarchical management, including 11 modules, the personnel management (personnel files, personnel transfer), resource management (training, on-the-job training, resources), information release (information release, the picture show), department (profiles, intensive nursing specialty, diabetes care, blood purification nursing specialty, disinfection supplies, obstetric nursing, emergency nursing, operating room nursing and cancer care professionals Industry), online exam (exam course, question, question bank management, test management and test statistics and print setup), credit management (normal subjects, the common examinations, online examination results, browse records management and department ranking), training plan (training plan management, training plan ranking), data dictionary (department information, the types of dictionary and data dictionary), training survey (training survey introduction, training survey management, training survey statistics and survey personnel statistics), bedside comprehensive ability (teacher setting, examination items, operation requirements, examination papers, examination statistics and examination audit) and authority management (authority type, management personnel). It can realize the automatic management of the whole process of nursing staff information, operation standards, operation assessment plan, operation assessment process, operation assessment results, statistical analysis (automatic display of test time and points deduction reason, an automatic summary of assessment results, assessment results batch export and review). (2) APP end: it contains 8 modules, including training resources, training plan, on-the-job training, online exam, notice and announcement, training survey, score check, and bedside exam score of operation skills.

Application process: The platform is equipped with 4 senior hospital administrators to conduct all-round supervision on the training and assessment of nursing staff in the hospital(the 4 administrators were involved in the whole process from the beginning of this study); In addition, excellent assessment teachers are selected from the whole school according to the comprehensive assessment results such as teaching years, working years, professional title, educational background, and operating skill level. After passing the assessment, permission to use the platform will be opened(Although the teachers participating in the training are different before and after the use of the information training platform, the inclusion criteria of the training teachers are the same before and after the use of the information training platform). Training process: teachers are assessed to enter the training evaluation system through natural coding, and select the corresponding test project from training resources. Then the selected teacher shall carry out a demonstration in accordance with the platform standard for the examinee. The next part is the grouping exercise, that is, every demonstration teacher will be in charge of a group of students and provide on-site guidance to the examinee (On average, each examinee completed one operation under the guidance of the demonstration teacher). After the demonstration teacher’s guidance, the examinee will practice by himself and be assessed within the prescribed time. Assessment process: The assessment teacher enters the training evaluation system, selects the corresponding assessment items, inputs the natural code of the examinee, clicks “Start” to enter the assessment and start timing, deducts the corresponding points according to the assessment standards, clicks “End” to stop timing, clicks “submit” to query the scores of the examinee and print the test papers in the management center, and then complete the project of all assessment personnel scores, error rate, and other real-time statistics. All methods were performed according to internet-based e-learning guidelines [6] and clinical nursing operation training and assessment guidelines [7, 8].

The trial run of an information-based training and assessment platform for clinical nursing operation techniques in a tertiary level A general hospital in Shandong Province at the end of 2017. Since 2018, the platform has been officially launched, and the clinical operation skills training and assessment of all nurses in the hospital are carried out on this platform. Before 2018, the traditional paper version of the training assessment method, namely the assessment of the training teachers will be training all the problems on paper records, and finally the data for a one-by-one summary analysis, due to the time-consuming and labour-intensive nature of the method and the constraints of human resources, no standardized training on nursing operational skills has been adopted until 2018, namely, no information-based training platform for reference after the demonstration of the training teachers, the training object practices by itself, carries on the appraisal in the fixed time.

### Evaluation of the effect of information-based training and assessment platform for clinical nursing operation technology

In this study, a convenient cluster sampling method was used to evaluate the effectiveness of an information-based training and assessment platform for clinical nursing operation techniques. The inclusion criteria were: (1) registered nurses from a grade iii-a general hospital in Jinan, Shandong Province, China; (2) N 1 ~ N 4 level for nurses and (3) completion of training assessment within a specified period time. Exclusion Criteria: (1) trainee nurses or advanced nurses; (2) new nurses (because new nurses have not yet adopted the information platform for training assessment) ; (3) those with fewer participants in special skills operations, such as pregnancy, etc. Although the data collection method of this study was retrospective, all the data in the past years of this study can be consulted in the database. The paper version data before 2018 were timely entered into the database by two researchers after the completion of the assessment, and all the evaluation data can be automatically uploaded to the database after the information-based training and assessment platform was adopted. Meanwhile, since the beginning of this study, all data were collected and managed by two main researchers. In addition, these two researchers were also responsible for the homogenization evaluation of all trainers to ensure the consistency of training assessment standards, so as to ensure the reliability and validity of data results. The main evaluation indicators include training and assessment of the two aspects of assessment results. (1) Number of training and assessment: by the end of 2020, the number of nursing personnel training and assessment on the operation skills of the platform since its operation for three years will be summarized and compared with the number before its use; (2) Comparative Analysis of Assessment Scores: In this study, we compared the performance of the same nurse with the same nursing technique, the same level with the same nursing technique before and after the use of the information platform. The study was carried out according to the relevant guidelines for training and assessment of nursing operational skills [7, 8], and the training teachers all passed the training and assessment of hospital-level administrators, after reaching the homogeneity of grasping all the standards, only then may carry on the training appraisal work to the nurse.

### Data analysis

In this study, SPSS 25.0 was used to analyze the data, the classified data were described by frequency and composition ratio, If the measurement data conform to the normal distribution, the descriptive analysis was carried out by Mean(SD), and the comparative analysis was carried out by t-test or analysis of variance; If the results did not fit a normal distribution, M (P25, P75) was used for descriptive analysis, and rank sum test was used for comparative analysis. The chi-square test was used to analyze the error rates recorded in the two-year operation evaluation.

## Results

### Number of training and assessment

Since the application of the information-based training and assessment platform began in 2018, a total of 12,619 nursing operation skills training and a total of 11,986 assessments have been completed from 2018 to 2020 (Table 1). Significantly more nursing staff operational skills assessment visits in 2018 to 2020 compared to 2016 and 2017 (Table 2).

**Table 1:** Summary of training and assessment attendance from 2018 to 2020 (*n*)

Hierarchy	2018		2019		2020	
	training	assessment	training	assessment	training	assessment
N1	292	287	260	254	238	238
N2A	238	233	279	272	242	242
N2B	402	390	242	236	260	260
N2C	656	300	396	386	221	221
N3A	440	440	314	304	652	652
N3B	374	244	272	272	604	604
N3C	420	384	492	492	172	172
N3D	480	430	382	382	679	679
N3E	492	492	344	344	247	247
N4	982	982	1128	1128	419	419
Total	4776	4182	4109	4070	3734	3734

**Table 2:** Summary of assessment attendance from 2016 to 2020

Hierarchy	2016	2017	2018	2019	2020
N1	482	270	287	254	238
N2	1480	1312	923	894	723
N3	501	843	1990	1794	2354
N4	490	632	982	1128	419
Total	2953	3057	4182	4070	3734

### Effects of training and assessment

#### Comparative analysis of the same nurses; performance in the same operation

The results of nasal feeding, blood transfusion, and micropump before and after using the information platform in 2017 and 2018 of the same nurses were compared and analyzed. Since none of the scores matched the normal distribution (related material K-S normality test 1), a comparative analysis of the two-year scores was performed using the Wilcoxon Mann-Whitney rank-sum test. Before and after using the information-based training and assessment platform, there was statistical significance in the score distribution of blood transfusion and micro-pump operation (*p* < 0.05), but there was no statistical significance in the score distribution of nasal feeding (*p* > 0.05) (Table 3).

**Table 3:** Comparative analysis of the same nurses’ performance in the same operation

year	Nasal feeding		Blood transfusion		Micro pump	
	*n*	M(*P*_25_, *P*_75_)	*n*	M(*P*_25_, *P*_75_)	*n*	M(*P*_25_, *P*_75_)
2017	183	90.00(87.00,92.50)	195	90.00(87.50,92.50)	218	89.50(85.50,93.00)
2018	142	91.00(88.00,93.00)	118	92.75(90.50,94.50)	185	93.00(90.50,94.88)
*Z*		-1.733		-5.264		-7.456
*P*		0.083		< 0.001		< 0.001

Analysis of technical errors in blood transfusion and micropump operation is shown in the supplementary material. A comparative analysis of the operation errors of blood transfusion and micro pump before and after the use of the information platform showed that the error rates of four problems in the transfusion operation after the use of the information platform were significantly lower than those before the use of the information platform (*P* < 0.05). However, the error rate of timeout in operation time was significantly higher than before (*P* < 0.05)(Table 4). After the use of the information platform, the error rates of a non-standard check, incomplete explanation to patients, and non-standard use of micropump were significantly lower than before (P < 0.05). There was no significant difference between before and after the non-standard extraction method (*P* > 0.05), but the error rate of timeout in operation time increased after the use of the information platform (Table 5).

**Table 4:** Comparative analysis of operational errors of blood transfusion before and after using nursing Operation training information Platform

Item	2017(*n/%*)	2018(*n/%*)	χ2	*P*
Operation check is not standard	407(52.17)	92(19.49)	131.075	< 0.001
Execution form signature and time are not standard	323(55.21)	36(10.17)	189.487	< 0.001
Exhaust light check for bubbles	236(60.51)	55(23.30)	81.824	< 0.001
Operation timeout	157(80.51)	113(95.76)	14.427	< 0.001
The disinfection technique is not correct	144(73.85)	56(47.46)	22.191	< 0.001

Note: A total of 4 checks are required in this operation, so the total number of checks in 2017 is 195 * 4 = 780, and the total number of checks in 2018 is 118 * 4 = 472. Three checks are required to execute a single signature in the operation, thus, the total number of signatures in 2017 was 195 * 3 = 585, and the total number of signatures in 2018 was 118 * 3 = 354; the total number of exhaust-to-light inspections in 2017 was therefore 195 * 2 = 390, with 2 in-operation exhaust-to-light inspections; In 2018, the total number of exhaust light checks was 118 * 2 = 236

**Table 5:** Comparative analysis of operational errors of micro pump before and after using nursing Operation training information Platform

Item	2017(*n/%*)	2018(*n/%*)	χ2	*P*
Operation check is not standard	250(38.22)	70(12.61)	101.202	< 0.001
Incomplete explanation to patients	113(51.83)	32(17.30)	51.279	< 0.001
The use of micro pump is not standard	106(48.62)	50(27.03)	19.674	< 0.001
The extraction method is not standard	84(38.53)	52(28.11)	4.864	0.027
Operation timeout	82(37.61)	127(68.64)	38.606	< 0.001

Note: A total of 3 checks are required in this operation, so the total number of checks in 2017 is 218 * 3 = 654, and the total number of checks in 2018 is 185 * 3 = 555;

### Comparative analysis of the same operation results at the same level

The operational results of N1 level nurses who performed nasal feeding, CPR, airway balloon, and electric defibrillation during 2016–2020 were collected and analyzed. Because the results of nasal feeding did not conform to the normal distribution, the descriptive analysis was performed by M (P25, P75), and the comparative analysis was performed by Kruskal Wallis rank sum test, Mean(SD) was used for descriptive analysis, and analysis of variance was used for comparative analysis (related material K-S normality test 2). From 2016 to 2020, the scores for nasal feeding, CPR, and air balloon for N1 nurses increased significantly after using the information training and assessment platform (*P* < 0.05), but there was no significant change in the scores for electric defibrillation (*P* > 0.05) (Table 6).

**Table 6:** Summary and analysis of results of N1 nurses from 2016 to 2020

year	Nasal feeding		CPR		breathing bag		Electrical shock	
	*n*	M(*P*_25_, *P*_75_)	*n*	Mean(SD)	*n*	Mean(SD)	*n*	Mean(SD)
2016	205	91.00(88.00,94.50)	64	91.71(1.82)	69	92.30(2.70)	74	90.63(2.35)
2017	133	91.50(89.50,93.50)	43	91.63(3.47)	44	91.17(3.01)	44	90.34(2.70)
2018	145	93.00(90.00,95.00)	49	91.67(2.71)	47	93.57(2.84)	51	90.76(3.46)
2019	126	93.50(91.38,95.00)	42	92.74(2.60)	42	93.03(3.63)	43	90.85(2.93)
2020	119	93.50(92.00,95.00)	61	94.17(2.02)	38	93.59(3.42)	42	91.67(2.60)
*H/F*		72.948		11.379		4.867		1.329
*P*		0.000		< 0.001		0.001		0.260

## Discussion

Clinical nursing skills training assessment at home and abroad is still mainly in the form of print, there are many shortcomings, such as the result analyzed time-consuming, waste paper, it is difficult to identify the handwriting, and so on, this study developed by nursing operation skill training examination platform based on the information terminal is going with the development of The Times, the results show that the platform can not only improve operation test scores, But also reduce the incidence of errors, and can significantly save the analysis of performance time, improve work efficiency, improve overall performance.

### Information training and assessment platform of clinical nursing operation technology can significantly improve work efficiency

From 2018 to 2020, a total of 12,619 people were trained in nursing operation skills, and a total of 11,986 people were assessed (in 2019, due to a slight decrease from 2018, the number of people was reduced in 2020 due to the impact of COVID-19). Compared with 2018–2020 and 2016/2017, the number of trainees increased significantly (before 2018, due to the time and effort of data analysis and collation, only the evaluation of each operation was carried out, and the training was not standardized enough, so the specific training number was not counted). It can be seen that under the same human resources, Fully reflecting the information-based terminal training assessment system can significantly improve work efficiency. According to the findings of Ying et al. [9] in 2018, Information Technology can improve nurses’ work efficiency and reduce the incidence of adverse nursing events, which is consistent with the findings of this study. This information platform has the following advantages: The APP end can input natural code corresponding to the examinee, completely avoiding the occurrence of the same name and other phenomena; Automatic timing after the operation test, automatic total score after the operation; Management center computer terminal can realize the management of the whole nursing staff and the operations of appraisal criteria and video recording, the evaluation process of regulation, keep records, evaluation results of the assessment results in bulk export, examine error summary analysis, etc. The whole process of management, so compared with the traditional print training evaluation, can save a lot of performance calculation and entry, and analysis procedures, Significantly improve work efficiency; In addition, the training and evaluation platform based on the information terminal can input the natural code at any time to review the results, export the results at any time to measure the homogeneity of the evaluation teacher, and monitor whether the scoring standards of each invigilator are consistent at any time, to ensure the fairness and justice of each examinee’s training and evaluation.

### The information training and assessment platform of clinical nursing operation technology can significantly improve the overall performance

This study showed that the scores of transfusion and micropump in 2017 were significantly lower than those in 2018 (P < 0.05), and the error rates of non-standard operation verification and non-standard execution of single signature were significantly lower (P < 0.05). Moreover, from 2016 to 2020, the scores of the three nursing operation examinations of N1 level nurses, namely, nasal feeding, CPR, and air balloon, all increased significantly after the use of the information-based training and assessment platform. Therefore, the scores of the same level nurses and the same level nurses all improved significantly (because the nursing operation items of the training and assessment of the nurses at other levels from 2016 to 2020 are different and can’t be compared and analyzed, so only the scores of the nurses at N1 level are compared), this finding is consistent with the findings of Voutilainen et al. [10]. The meta-analysis of e-learning is consistent, an e-learning method resulted in test scores that were, on average, five points higher than a conventional method on a 0-100 scale. However, in this study, the timeout error rate of the two operations in 2018 was significantly higher than that in 2017 (P < 0.05), which may be related to the fact that nurses paid too much attention to the details of the operation or were not skilled enough in the operation. Therefore, in the future, we should pay attention to the time while paying attention to the details of the operation. Although based on information terminal for examination and assessment of nurse training platform can promote appraisal result, but not to reform training method innovation, continue with the teacher training in the form of demonstration, research has shown that training methods affect performance is critical, with the deepening of the connotation of nursing, nursing training method is also in the continuous improvement innovation, All kinds of information-based clinical teaching methods have emerged at home and abroad. For example, virtual reality simulation [3]/ E-learning [11, 12]/ digital technology [13]/ information and communication technology [14, 15] etc. Although it can improve the training effect, it is affected by many factors, such as learning style [16], which still needs to be further verified by clinical practice, especially for disability education [17]. But the effectiveness of information training and assessment platform for nurses in a continuing education context remains unknown regarding how the learning can be transferred to change practice and affect patient outcomes [12].

## Conclusion

Based on the information terminal training and assessment can realize the whole process management of clinical nursing operation technology training and assessment, compared with the traditional training and assessment method, can improve the work efficiency and assessment results, is a very practical and convenient clinical training and assessment method.

## Limitation

This study has the following defects: (1) this study was only implemented in a tertiary level A general hospital, and was a single-center study; (2) This study is a retrospective study, and randomized controlled trials can be carried out in multiple centers in the future to better verify the reliability of the results.

## Electronic supplementary material

Below is the link to the electronic supplementary material.

Supplementary Material 1

## Data Availability

Data materials are real and available. Chunlan Liu should be contacted if someone wants to request the data from this study.
