# Supplementary material for: Establishment and application of information-based training and assessment platform for clinical nursing operation technology
Source: BMC Nurs. 2022 Oct 18;21:282. doi: 10.1186/s12912-022-01053-3 (PMC9578263; doi:10.1186/s12912-022-01053-3)
Supplement: Supplementary file 1 — Supplementary Material 1 [file 12912_2022_1053_MOESM1_ESM.docx]

Table1 Error analysis of micro pump operation technology in 2017 and 2018

| 2017 ( *n* = 162 ) (n/%) | | | 2018 ( *n* = 188) (n/%) | | |
| --- | --- | --- | --- | --- | --- |
| Operation Check is not standard | 250 | 154.32 | Operation timeout | 127 | 67.55 |
| Failure to assist patient in comfortable position | 118 | 72.84 | Air in syringe and extension tube is not emptied | 118 | 62.77 |
| Incomplete explanation to patients | 113 | 69.75 | Nonstandard disinfection | 116 | 61.70 |
| The use of micro pump is not standard | 106 | 65.43 | Poor sterility concept | 81 | 43.09 |
| The extraction method is not standard | 84 | 51.85 | Operation check is not standard | 70 | 37.23 |
| Operation timeout | 82 | 50.62 | Unobstructed infusion was not observed | 68 | 36.17 |
| Poor sterility concept | 67 | 41.36 | The extraction method is not standard | 52 | 27.66 |
| Execution form signature is not standard | 64 | 39.51 | The use of micro pump is not standard | 50 | 26.60 |
| Unobstructed infusion was not observed | 58 | 35.80 | Place the ampoule in a sterile tray and paste the label immediately | 48 | 25.53 |
| Nonstandard disinfection | 57 | 35.18 | Wrong connection between extension tube and syringe | 40 | 21.28 |

Table 2 Error analysis of transfusion techniques in 2017 and 2018

| 2017 ( n = 162 ) (n/%) | | | 2018 ( n = 188) (n/%) | | |
| --- | --- | --- | --- | --- | --- |
| Operation check is not standard | 407 | 187.56 | Operation timeout | 113 | 94.96 |
| Execution form signature and time are not standard | 323 | 148.85 | Operation check is not standard | 92 | 77.31 |
| Exhaust light check for bubbles | 236 | 108.76 | Not proficient in operation | 67 | 56.30 |
| The adjustment of dripping speed is not standard | 161 | 74.19 | Poor sterility concept | 62 | 52.10 |
| Operation timeout | 157 | 72.35 | Disinfection bag plug is not standard | 56 | 47.06 |
| Incorrect disinfection and plump cotton swabs | 144 | 66.36 | Exhaust light check for bubbles | 55 | 46.22 |
| No transfusion reaction was observed | 132 | 60.83 | Execution form signature and time are not standard | 36 | 30.25 |
| Treatment materials are not standardized | 121 | 55.76 | The area of skin disinfected is incorrect | 32 | 26.89 |
| Did not inform the custodian of adverse blood transfusion reactions and precautions | 105 | 48.39 | The blood was not shaken evenly and the temperature was tested | 29 | 24.37 |
| The puncture site was not examined | 57 | 42.40 | The blood transfusion form does not indicate the end time | 25 | 21.01 |
